# Supplementary material for: mRNA-LNP vaccines against hepatitis B virus induce protective immune responses in preventive and chronic mouse challenge models
Source: Mol Ther. 2025 Jun 14;33(9):4156–74. doi: 10.1016/j.ymthe.2025.06.027 (PMC12432868; doi:10.1016/j.ymthe.2025.06.027)
Supplement: Supplementary file 1 [file mmc1.pdf]

YMTHE, Volume *33*

Supplemental Information

mRNA-LNP vaccines against hepatitis B virus induce protective immune responses in preventive and chronic mouse challenge models

María José Limeres, Rocio Gambaro, Malin Svensson, Silvia Fraude-El Ghazi, Leah Pretsch, Daniel Frank, German A. Islan, Ignacio Rivero Berti, Matthias Bros, Ying K. Tam, Hiromi Muramatsu, Norbert Pardi, Stephan Gehring, and Maximiliano L. Cacicedo

Supplemental Material

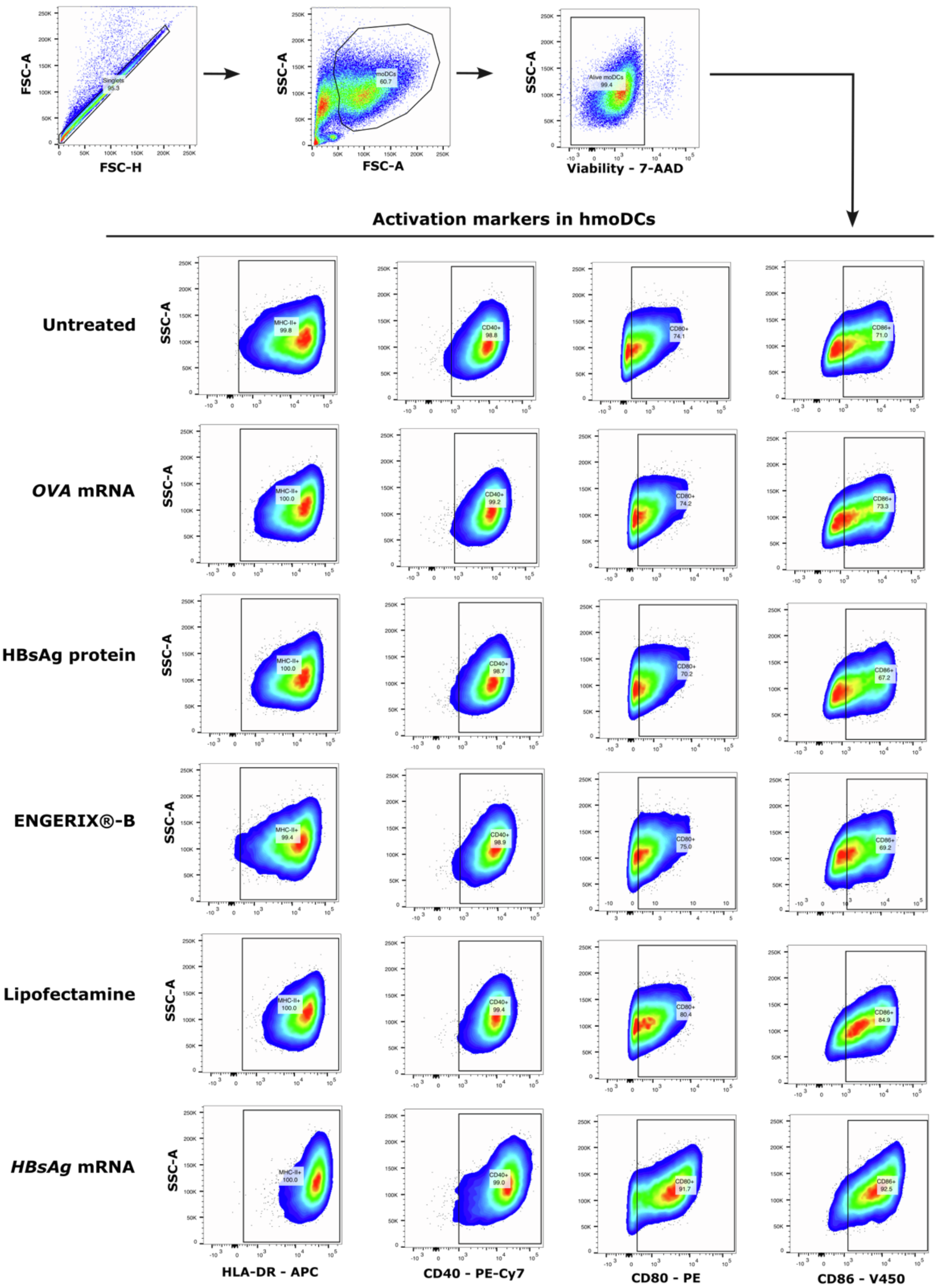

Figure S1. Gating strategy of the hmoDC transfection. Flow cytometry dot plots of the unstimulated and transfected hmoDCs with *HBsAg* mRNA to study their activation through HLA-DR, CD40, CD80 and CD86 surface markers. OVA mRNA, HBsAg protein, ENGERIX®-B and Lipofectamine served as controls.

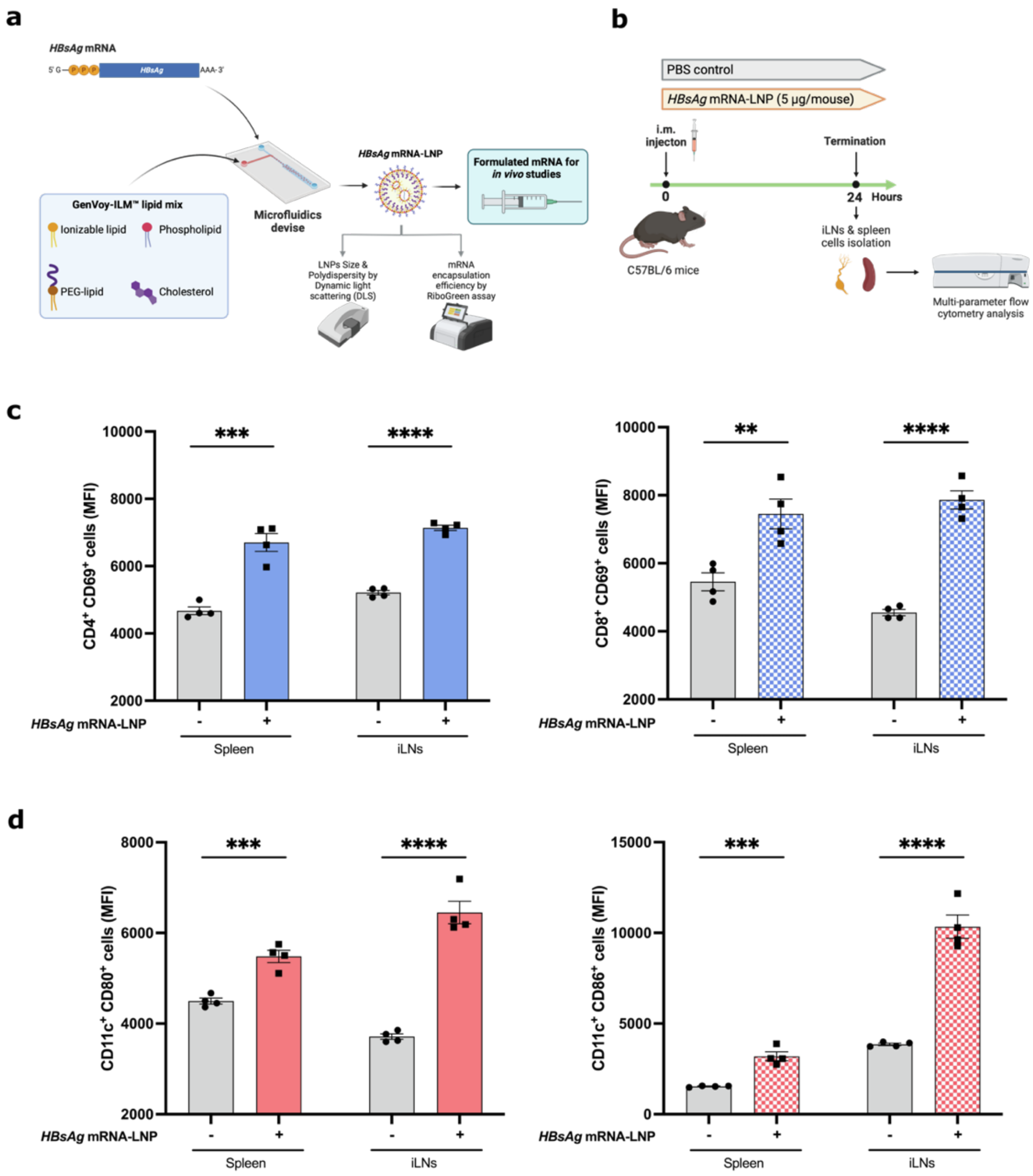

Figure S2: *HBsAg* mRNA formulated in LNP induces early activation of DCs and T cells in secondary lymphoid organs. (a) Schematic representation of mRNA-based vaccine preparation. *HBsAg* mRNA was formulated in LNP, and mRNA-LNP were then characterized. (b) Experimental design. Mice were vaccinated i.m. with 5 µg *HBsAg* mRNA-LNP. Spleen and inguinal lymph nodes were dissected for evaluation 24 hours after injection. Expression of early activation markers (c) CD69 by $CD4^+$ and $CD8^+$ T cells, and (d) CD80 and CD86 by DCs isolated from the spleen and lymph nodes were compared to the expression of cells derived from the PBS control group. Data are the means ± SEM for animal cohorts (4 mice/group). Significantly different from the control group: **p< 0.01, ***p< 0.001; ****p <0.0001 (two-tailed Student's *t*-test).

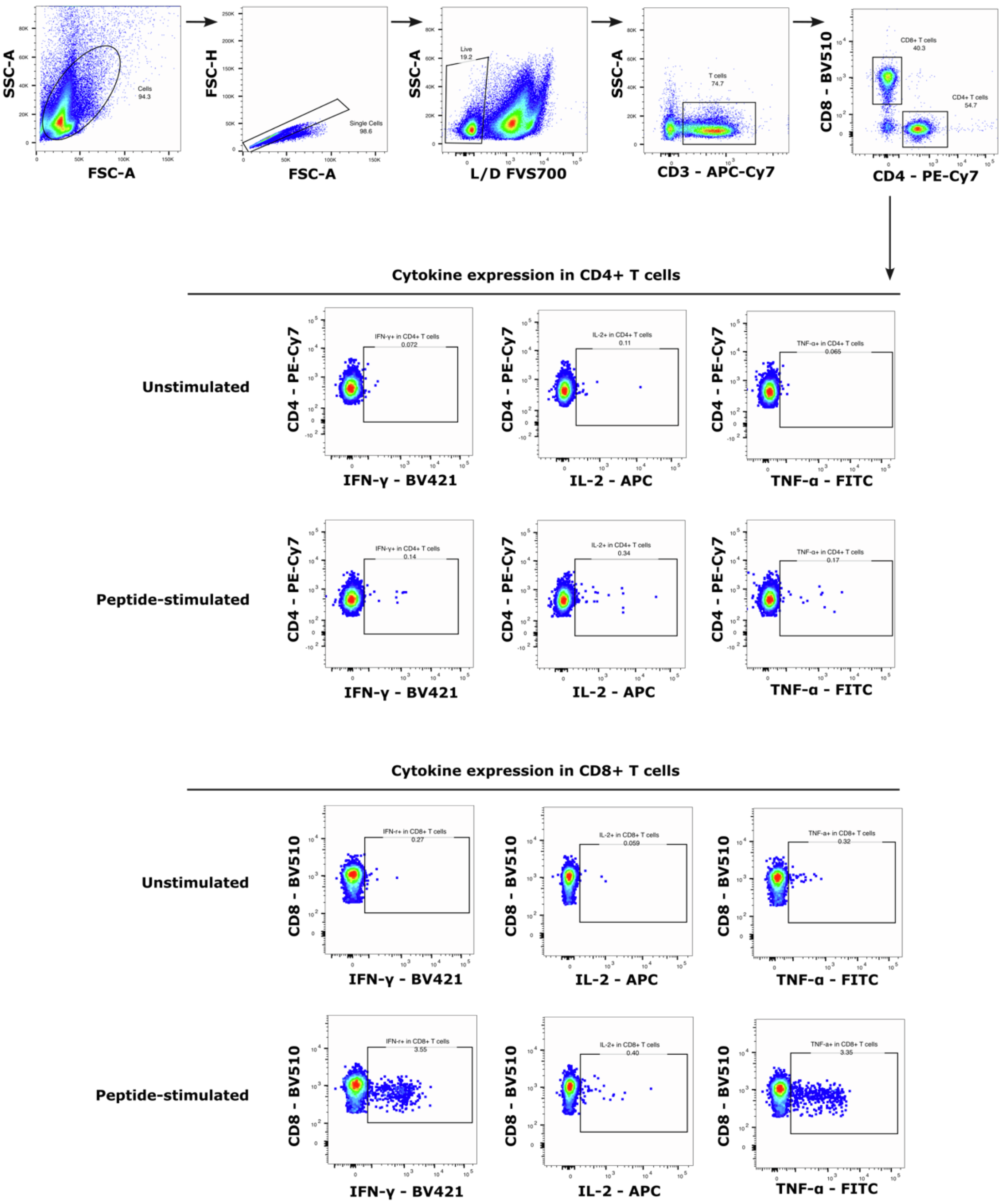

Figure S3. Gating strategy of the intracellular cytokine staining. Flow cytometry dot plots of the unstimulated and peptide-stimulated splenocytes to study the specific $CD4^+$ and $CD8^+$ T cell responses in immunized mice.

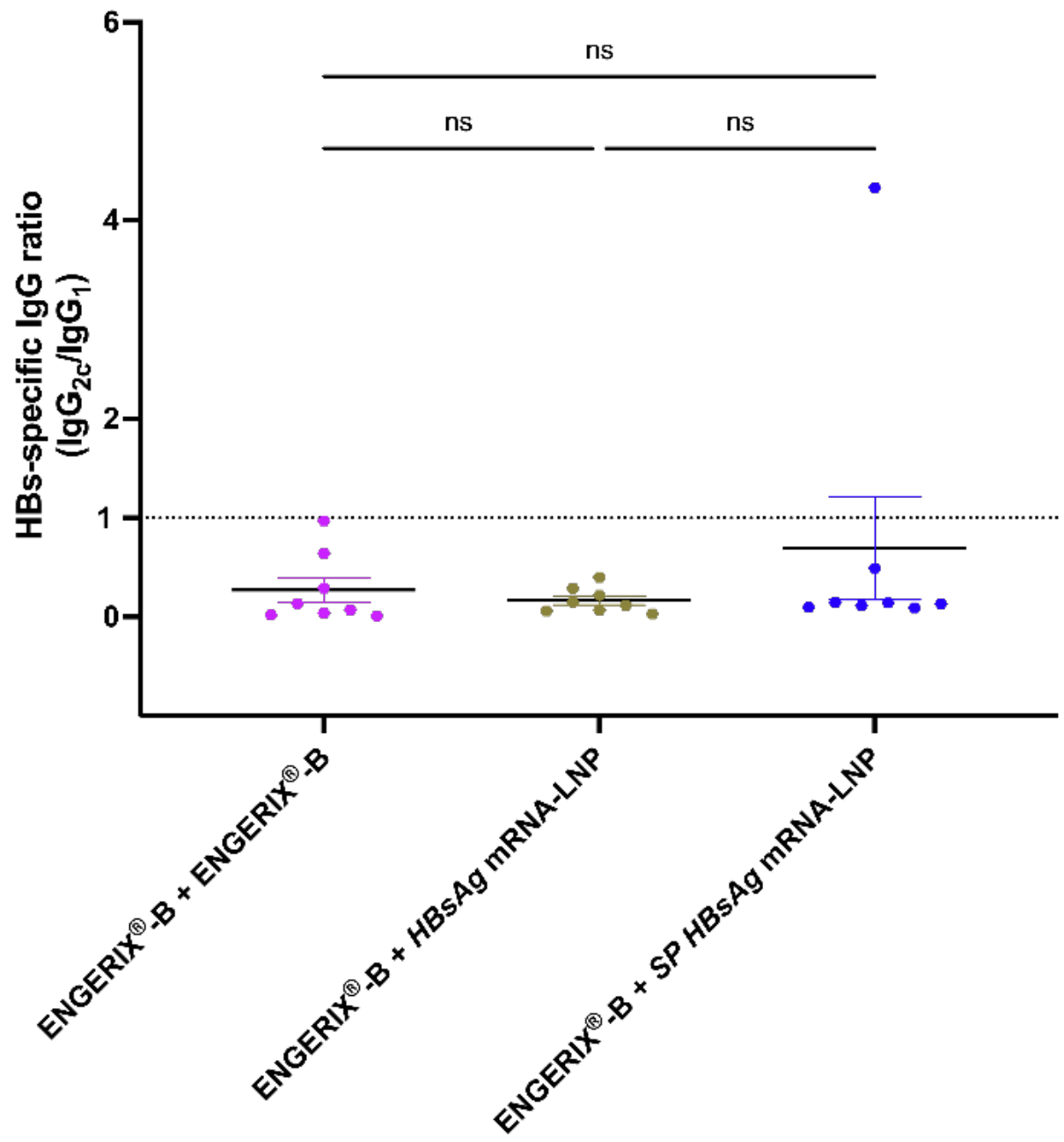

Figure S4: Anti-HBsAg IgG subtypes antibodies from mice immunized with the heterologous immunization schedule. IgG2c/IgG1 ratio at termination (d42). Data are the means ± SEM for animal cohorts (8 mice/group). Significance tested compared to the other groups: no significant differences (ns) (one-way ANOVA, Tukey's multiple comparisons test).

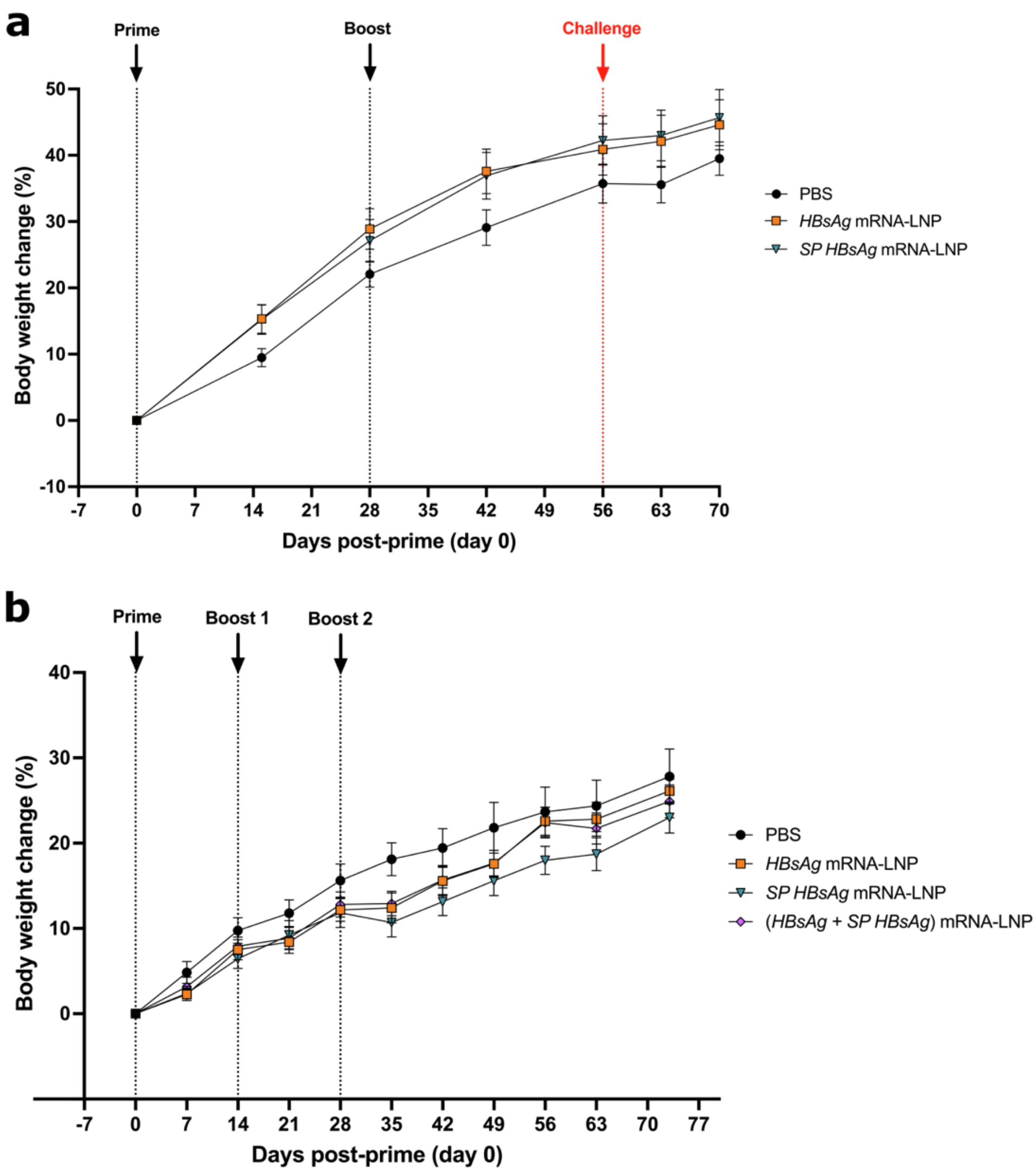

Figure S5. Body weight change in immunized mice. Evaluation of the reactogenicity by measuring the percentage of the body weight change in rAAV8-1.3HBV challenged mice (a) and in CHB mice (b).

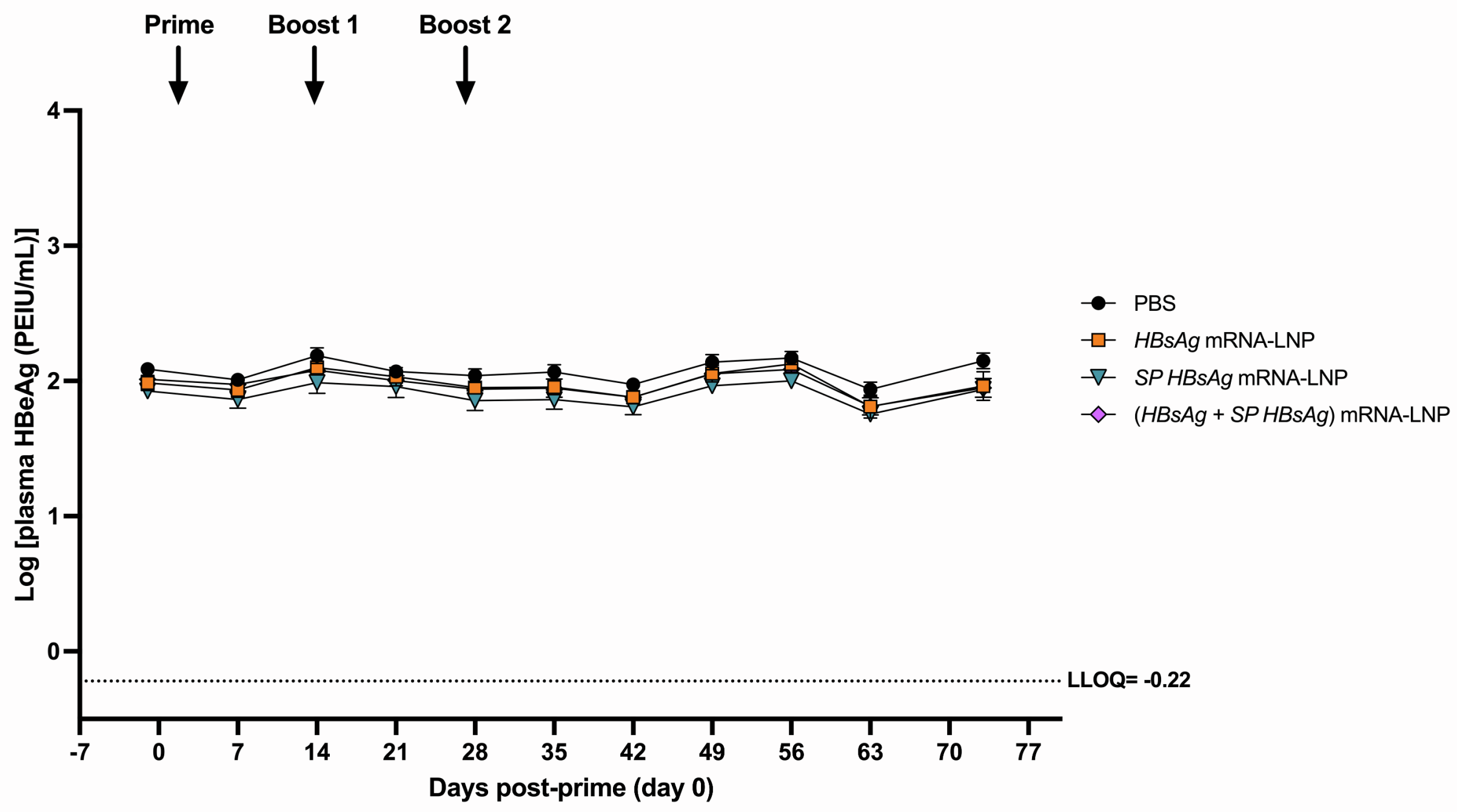

Figure S6. Measurement of plasma HBeAg in CHB mice. Evaluation of plasma HBeAg levels in HBV-carrier mice during the immunization schedule and follow-up until day 73 post-prime administration.

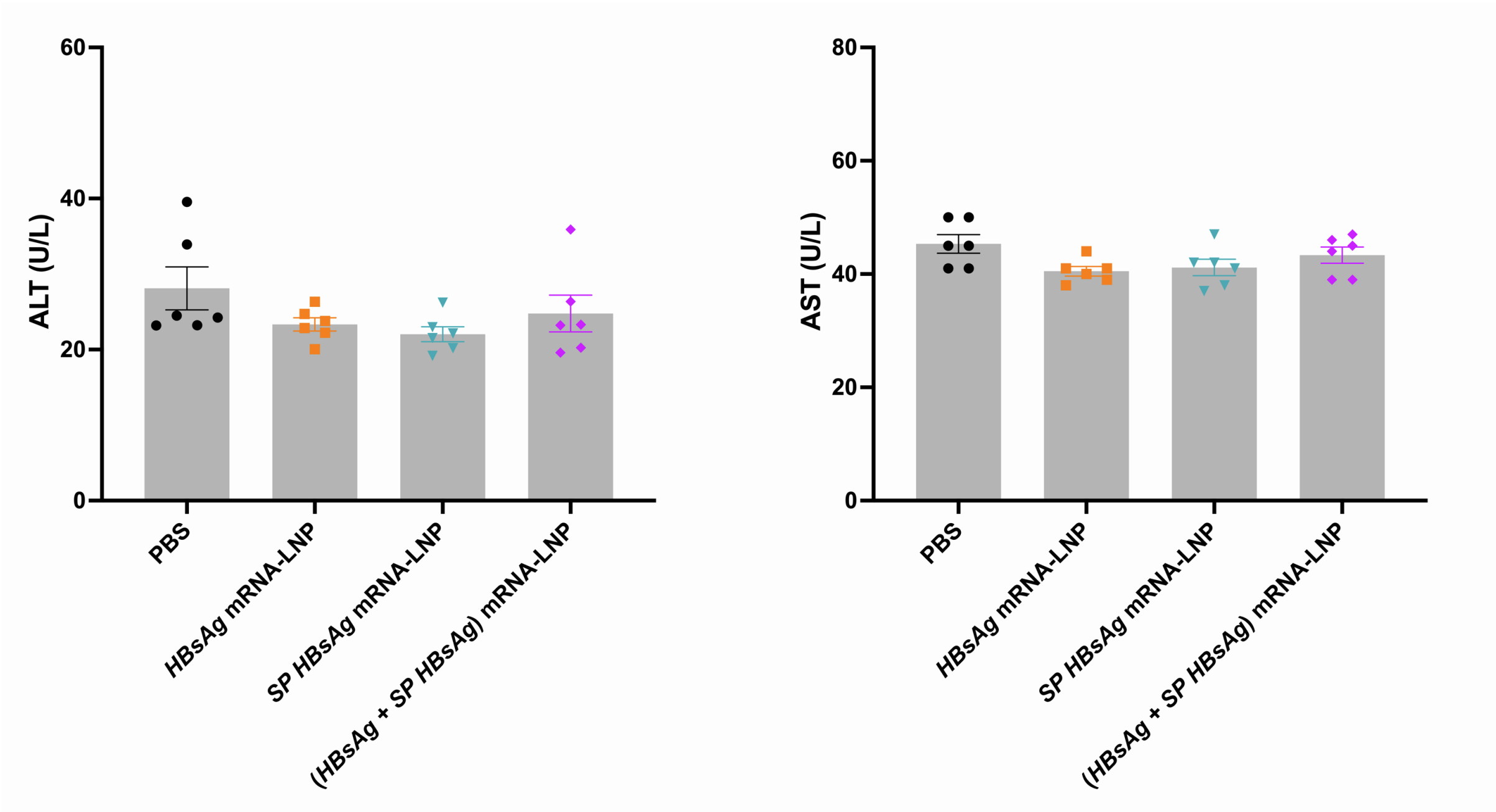

Figure S7. Evaluation of plasma transaminases concentration in CHB mice. Measurement of plasma ALT and AST levels in HBV-carrier mice at day 73 post-prime administration. Data are the means ± SEM for animal cohorts (6 mice/group). Significance tested compared to the other groups (one-way ANOVA, Tukey's multiple comparisons test).

Table S1: List of antibodies used for flow cytometry analysis.

Antibody	Fluorochrome	Reference	Clone	Dilution
**Activation markers of transfected/stimulated hmoDCs**				
HLA-DR	APC	559866	G46-6	1/10
CD86	BD Horizon^{TM} V450	560357	2331 (FUN-1)	1/25
CD80	PE	557227	L307.4	1/10
CD40	PE-Cy^{TM}7	561215	5C3	1/25
***In vivo* DC and T cell activation**				
CD8a	Super Bright^{TM} 436	62-0081-82	53-6.7	1/125
CD3	eFluor^{TM} 506	69-0032-82	17A2	1/60
CD80	PerCP-eFluor^{TM} 710	46-0801-82	16-10A1	1/200
CD4	FITC	553047	L3T4	1/800
CD69	PE-Cy^{TM}7	552879	H1.2F3	1/80
CD11c	PE	117308	N418	1/100
CD86	APC	105012	GL-1	1/100
**Intracellular cytokine staining**				
IL-2	APC	503810	JES6-5H4	1/80
IFN-γ	BD Horizon^{TM} BV421	563376	XMG1.2	1/100
TNF-α	FITC	11-7321-82	MP6-XT22	1/200
CD8α	BD Horizon^{TM} BV510	563068	53-6.7	1/200
CD3ε	APC-Cy^{TM}7	100330	145-2C11	1/40
CD4	PE-Cy^{TM}7	563933	GK1.5	1/200
